# Cuticular Wax Modification by *Epichloë* Endophyte in *Achnatherum inebrians* under Different Soil Moisture Availability

**DOI:** 10.3390/jof8070725

**Published:** 2022-07-12

**Authors:** Zhenrui Zhao, Yawen Ju, Mingzhu Kou, Mei Tian, Michael John Christensen, Xingxu Zhang, Zhibiao Nan

**Affiliations:** 1 State Key Laboratory of Grassland Agro-Ecosystems, Key Laboratory of Grassland Livestock Industry Innovation, Ministry of Agriculture and Rural Affairs, College of Pastoral Agriculture Science and Technology, Lanzhou University, Lanzhou 730020, China; zhaozhr16@lzu.edu.cn (Z.Z.); juyw17@lzu.edu.cn (Y.J.); koumzh18@lzu.edu.cn (M.K.); zhibiao@lzu.edu.cn (Z.N.); 2 Huaiyin Institute of Agricultural Sciences of Xuhuai Region in Jiangsu, Huai’an 223001, China; 3 Institute of Horticulture, Ningxia Academy of Agricultural and Forestry Sciences, Yinchuan 750002, China; 4 Grasslands Research Centre, Private Bag 11-008, Palmerston North 4442, New Zealand; mchristensenpn4410@gmail.com

**Keywords:** *Epichloë gansuensis*, *Achnatherum inebrians*, soil moisture availability, cuticular wax, gas chromatography−mass spectrometry, transcriptome analysis

## Abstract

The cuticular wax serves as the outermost hydrophobic barrier of plants against nonstomatal water loss and various environmental stresses. An objective of this study was to investigate the contribution of the mutualistic fungal endophyte *Epichloë gansuensis* to leaf cuticular wax of *Achnatherum inebrians* under different soil moisture availability. Through a pot experiment and gas chromatography−mass spectrometry (GC−MS) analysis, our results indicated that the hydrocarbons were the dominant components of leaf cuticular wax, and the proportion of alcohols, aldehydes, amines, and ethers varied with the presence or absence of *E. gansuensis* and different soil moisture availability. Amines and ethers are unique in endophyte-free (EF) *A. inebrians* plants and endophyte-infected (EI) *A. inebrians* plants, respectively. By transcriptome analysis, we found a total of 13 differentially expressed genes (DEGs) related to cuticular biosynthesis, including *FabG*, *desB*, *SSI2*, *fadD*, *BiP*, *KCS*, *KAR*, *FAR*, and *ABCB1*. A model is proposed which provides insights for understanding cuticular wax biosynthesis in the association of *A. inebrians* plants with *E. gansuensis.* These results may help guide the functional analyses of candidate genes important for improving the protective layer of cuticular wax of endophyte-symbiotic plants.

## 1. Introduction

Plants, unlike animals, inevitably withstand various biotic and abiotic stresses at the sites of growth, such as diseases, pests, drought, salinity, extreme temperatures, and heavy metals. As a major factor affecting plant growth and production, drought alone causes more annual losses in crop yields than all pathogens combined [1]. Droughts of the future are likely to be more frequent, severe, and longer-lasting than in recent decades [2]. To increase tolerance in harsh situations, plants create beneficial symbioses with a range of organisms, including mycorrhizal fungi, nitrogen-fixing bacteria, and endophytic fungi [3,4,5].

Endophytic fungi of the genus *Epichloë* have been found to form mutualistic associations with many cool season grasses which can enhance survival of host plants through protection against abiotic and biotic stresses [6,7,8]. Nearly 100% of *A. inebrians* plants, a perennial bunchgrass in the arid and semi-arid grasslands of northwest China, are symbiotic with either *E. gansuensis* [9] or *E. inebrians* [10]. According to the previous studies, the association of *A. inebrians* plants with *E. gansuensis* can improve the tolerance to diseases [11], pests [12], low temperature [13], heavy metals [14], and drought [15].

Cuticular wax deposition and composition is closely related to drought resistance and yield in plants, since cuticular wax serves as the outermost hydrophobic barrier against nonstomatal water loss and various environmental stresses [16]. Cuticular waxes, complex mixtures, predominantly contain very-long-chain fatty acids (VLCFAs), hydrocarbons, alcohols, aldehydes, ketones, esters, triterpenes, sterols, and flavonoids [17]. The biosynthesis of cuticular wax is accomplished in two steps: C16 and C18 fatty acids are extended to the VLCFAs by fatty acid elongase complexes through an iterative cycle of four reactions: two-carbon elongation of fatty acyl-Coenzyme A (CoA) by 3-ketoacyl-CoA synthase (KCS), reduction of 3-ketoacyl-CoA by 3-ketoacyl-CoA reductase (KCR), dehydration of 3-hydroxyacyl-CoA by 3-hydroxyacyl-CoA dehydratase (HCD), and reduction of trans 2,3-enoyl-CoA by trans 2,3-enoyl-CoA reductase (ECR), and then each VLCFA is converted into wax components by decarbonylation and acyl reduction pathways in the endoplasmic reticulum (ER). The decarbonylation process mediates the formation of aldehydes, secondary alcohols, alkanes, and ketones, whereas the acyl reduction pathway is responsible for the formation of primary alcohols and wax esters [18]. Finally, various wax compounds are transported from the ER to the plasma membrane (PM), moved through the PM, and translocated across the cell wall to the cuticle by lipid transfer proteins (LTPs) and adenosine triphosphate (ATP)-binding cassette (ABC) transporters [19]. The composition and proportion of cuticular wax varies greatly among plant species, including the Poaceae grasses [17]. For example, primary alcohols are the most abundant components in cuticle wax of the leaves of wheat (*Triticum aestivum*) [20], maize (*Zea mays*) [21], barley (*Hordeum vulgare*) [22], *Aegilops tauschiic* [23], *Brachypodium distachyum* [24], and *Poa pratensis* [25], whereas in rice (*Oryza sativa*) leaves, wax mixtures are dominated by fatty acids [26]. Furthermore, cuticular wax deposition enhances drought resistance among many plant species, including tall fescue (*Festuca arundinacea*) [27], *Sorghum bicolor* [28], rice [29], barley [30], and maize [31]. Although regulatory mechanisms of cuticular wax biosynthesis are relatively less well-understood in grasses compared to *Arabidopsis thaliana*, several transcription factors involved in cuticular wax biosynthesis have been characterized. For instance, in maize leaves, there are at least 18 *Glossy* genes that can affect the synthesis and composition of cuticular wax [32]. The *OsWR1*, *OSWSL1*, and *DWA1* can activate cuticular wax biosynthesis to improve drought tolerance in rice [29,33,34]. However, studies on how endophytes influence the deposition, composition, and synthesis of cuticular wax under drought stress have not been reported. Our previous study suggested that the presence of an *Epichloë* endophyte decreased the fatty acid content of leaf epidermal wax of *A. inebrians* plants under different developmental stages at 1, 2, and 3 months [35].

In this study, we hypothesize that the association of *A. inebrians* plants with the endophyte (*E. gansuensis*) may result in changes in the composition of the cuticular wax under different soil moisture levels through influencing the expression of genes associated with key biosynthesis pathways. In order to test this hypothesis, we established endophyte-infected (EI) and endophyte-free (EF) *A. inebrians* plants in the greenhouse, and then set a challenge of three soil moisture availabilities, including drought stress. In addition, we measured the leaf cuticular wax composition and proportion by gas chromatography−mass spectrometry (GC−MS), as well as the expression levels of genes associated with the cuticular wax biosynthesis pathway by transcriptome analysis. In summary, an important foundation will be provided for future molecular studies and the regulatory mechanisms of *Epichloë* endophyte and drought stress on cuticular wax in *A. inebrians* plants based on these data.

## 2. Materials and Methods

### 2.1. Plant Materials and Experimental Treatments

Seeds were collected from a single EI and an EF *A. inebrians* plant grown in the experimental field of the College of Pastoral Agriculture Science and Technology, Yuzhong Campus of Lanzhou University (104°39′ E, 35°89′ N, Altitude 1653 m) in 2013. Seeds were stained with aniline blue and examined under light microscope at 40×power to look for fungal hyphae characteristic of *Epichloë* species to ensure that the infection rates of EI and EF plant seeds were 100% and 0%. Seed samples were maintained at 4°C and used for further study. A two-month pot experiment was carried out in a constant-temperature greenhouse (temperature: 26 ± 2 °C, moisture: 42 ± 2%) of the College of Pastoral Agriculture Science and Technology, Yuzhong Campus of Lanzhou University. At first, healthy-looking and well-filled seeds were sown in 120 pots (60 pots of EI plants and 60 pots of EF plants), with 3 seeds in each pot (diameter: 24 cm; height: 15 cm), filled with vermiculite (200 g) that had been sterilized in an oven at 180 °C for 2 h. These pots were watered appropriately and assigned at random to different positions regularly every day. After the second fully unfolded leaf appeared, Hoagland’s solution was applied to the pots every 7 days.

Twenty-five days after pot establishment, watering was ceased to reduce the soil moisture content of each pot to 15% relative saturated moisture content (RSMC), a ratio of actual soil moisture content to potential maximum soil water saturation. Five days after ceasing watering, that is, 30 days after planting, the 96 pots (48 with EI and 48 with EF) were thinned to a single plant. Three different soil moisture treatments were set up: 15% (drought, D), 30% (normal, N), and 60% (well-watered, W), with 16 pots of both EI and EF plants of each soil moisture content. During the trial, at 6 o’clock every evening, each pot was weighed and watered to maintain soil moisture content at 15%, 30%, or 60% RSMC, respectively. One month after the establishment of the soil moisture treatments, leaf samples from six random pots of each treatment were separately harvested, immediately frozen in liquid nitrogen, and maintained in a freezer at −80 °C for subsequent extraction of cuticular wax and transcriptome analysis.

### 2.2. Extraction of Cuticular Wax

Three replicate entire second leaf blades of each treatment were dipped in 10 mL of chloroform (Tianjin Guangfu Fine Chemical Research Institute, Tianjin, China) for 45 s to extract the total wax. As an internal standard, 20 μL n-tetracosane (1 μg μL^−1^, Aladdin, Shanghai, China) was added. The solution was concentrated to 1 mL under nitrogen. Then, 20 μL of N, O-Bis(trimethylsilyl)-trifluoroacetamide (GC Derivatization Reagent, ≥98.0%, Aladdin, Shanghai, China), and 20 μL pyridine (Standard for GC, >99.9%, Aladdin, Shanghai, China) were added, and the samples were maintained at 70 °C in a water bath for 1 h for derivatization. The solution was transferred to GC sample vials (1.5 mL) through organic filtration (0.45 μm) and blown dry under nitrogen. Finally, the samples were redissolved with 0.5 mL of chloroform for GC−MS analysis.

### 2.3. Chemical Analysis

Chemical analysis was performed by GC−MS (GCMS-QP2020, Shimadzu, Tokyo, Japan), coupled with a DB−1 MS capillary column (30 m length, 0.25 μm inner diameter, 0.25 μm film thickness, Agilent Technologies Inc., Palo Alto, CA, USA). Helium was used as a carrier gas at a flow rate of 1.2 mL min^−1^. The following other parameters were employed: inlet temperature, 280 °C; MS quadrupole temperature, 150 °C; transfer line temperature, 250 °C; ion source temperature, 230 °C; m z^−1^ range, 50−850. GC was carried out at the following temperature settings: 2 min at 50 °C, 40 °C min^−1^ to 200 °C, 2 min at 200 °C, 3 °C min^−1^ to 320 °C, and 30 min at 320 °C. Individual wax components were identified via the National Institute of Standards and Technology (NIST) 2017 library or comparing their mass spectra with the literature data. Single wax compounds were quantified against the internal standard by integrating the peak areas.

### 2.4. RNA Preparation and Transcriptome Sequencing

Total RNA was obtained from the six treatments, each with three biological replicates. The concentration and integrity of RNA was assessed using a Qubit^®^ RNA Assay Kit in Qubit^®^2.0 Flurometer (Life Technologies, Carlsbad, CA, USA) and an RNA Nano 6000 Assay Kit of the Agilent Bioanalyzer 2100 system (Agilent Technologies, Santa Clara, CA, USA).

Transcriptome sequencing was carried out by the Biomarker Technologies Company (Beijing, China). Libraries were generated using NEBNext^®^UltraTM RNA Library Prep Kit for Illumina^®^ (NEB, San Diego, CA, USA), and sequenced on an Illumina HiSeq 2500 platform. The library fragments were purified with an AMPure XP system (Beckman Coulter, Beverly, CA, USA).

### 2.5. Differentially Expressed Gene (DEG) Analysis

Fragments per kilobase per million mapped fragments (FPKM) values were calculated to evaluate the expression level of each gene. Differentially expressed gene (DEG) analysis was performed using DEseq2_EBseq, with a false discovery rate (FDR) ≤ 0.05 and fold change (FC) ≥ 2. Gene function was annotated based on the following databases: NR (NCBI non-redundant protein sequences); Pfam (protein family); KOG/COG/eggNOG (clusters of orthologous groups of proteins); Swiss-Prot (a manually annotated and reviewed protein sequence database); KEGG (Kyoto Encyclopedia of Genes and Genomes); and GO (gene ontology). GO enrichment analysis of the DEGs was performed by the GOseq R package based on a Kolmogorov–Smirnov test [36], and the statistical enrichment of DEGs in the KEGG pathway was determined by KOBAS software [37]. All raw sequences utilized in this study were deposited in Sequence Read Achieve (SRA) of the NCBI database under the accession number PRJNA748183.

### 2.6. Statistical Analysis

Differences in the proportion of cuticular wax under different endophyte status and soil moisture levels were tested using two-way analysis of variance (two-way ANOVA) by SPSS 22.0 (SPSS Inc., Chicago, IL, USA). Significant differences in the proportion of cuticular wax between EI and EF *A. inebrians* plants with the corresponding soil moisture content were examined by an independent-sample t-test. Fisher’s least significant differences (LSD) test was used to determine whether differences between means were statistically significant. In all tests, *p*-value < 0.05 was considered statistically significant. Data are the mean + standard error.

## 3. Results

### 3.1. Composition and Proportion of Leaf Cuticular Wax

**Figure 1:**
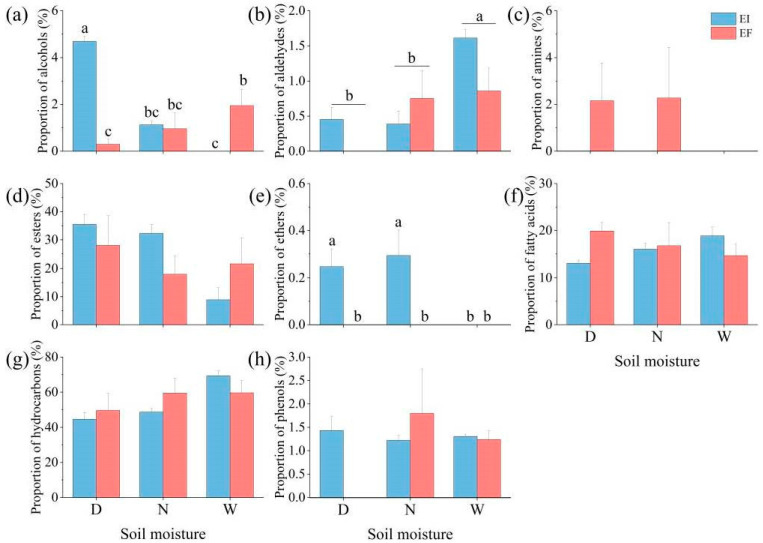
The proportion of alcohols (**a**), aldehydes (**b**), amines (**c**), esters (**d**), ethers (**e**), fatty acids (**f**), hydrocarbons (**g**), and phenols (**h**) of leaf cuticular wax in *Achnatherum inebrians* under the status of *Epichloë gansuensis* endophyte and different soil moisture content (D: drought, N: normal, W: well-watered, EI: endophyte-infected and EF: endophyte-free). Values are means, with standard error bars (*n* = 3). Columns with non-matching letters indicate a significant difference at *p* < 0.05.

The total leaf cuticular wax of *A. inebrians* plants under six treatments (DEI, DEF, NEI, NEF, WEI, and WEF) was collected individually via chloroform extraction. The composition of each wax extraction preparation was analyzed by GC–MS. A total of 59 different wax compounds were detected, which contained 44, 20, 31, 23, 34, and 39 compounds, respectively (Supplementary Tables S1–S6). In addition, the main wax components also varied among the six treatments, including alcohols (from minimum to maximum of 0–4.69%), aldehydes (from minimum to maximum of 0–1.61%), amines (from minimum to maximum of 0–2.28%), esters (from minimum to maximum of 8.88–35.47%), ethers (from minimum to maximum of 0–0.29%), fatty acids (from minimum to maximum of 13.12–19.89%), hydrocarbons (from minimum to maximum of 44.58–69.36%), and phenols (from minimum to maximum of 0–1.80%; Figure 1). Two-way ANOVA analysis showed that soil moisture content, the presence or absence of the *Epichloë* endophyte, and the interaction between the two factors had significant (*p* < 0.05) effects on the proportion of alcohols and ethers (Table 1). The proportion of alcohols of EI *A. inebrians* was significantly (*p* < 0.05) higher than that of EF *A. inebrians* under 15% soil moisture content. Under 30% soil moisture content, the status of *Epichloë* endophyte had no significant (*p >* 0.05) effects on the proportion of alcohols. Under 60% soil moisture content, the proportion of alcohols of EI *A. inebrians* was significantly (*p* < 0.05) lower than that of EF *A. inebrians*. With EI plants, the alcohol proportion at 15% soil moisture content was significantly (*p* < 0.05) higher than that at 60% soil moisture content. With EF plants, the alcohol proportion at 15% soil moisture content was significantly (*p* < 0.05) lower than that at 60% soil moisture content. At 15% and 30% soil moisture content, the ethers were found only in EI *A. inebrians*, and the proportion of ethers of EI *A. inebrians* was significantly (*p* < 0.05) higher than that of EF plants (Figure 1). Soil moisture content had significant effects on the proportion of aldehydes (Table 1). The aldehydes proportion of *A. inebrians* wax at 60% soil moisture content was significantly (*p* < 0.05) higher than that at 15% and 30% soil moisture content. However, soil moisture content, the presence or absence of the *Epichloë* endophyte, and the interaction between the two factors had no significant (*p* < 0.05) effects on the proportion of amines, esters, fatty acids, hydrocarbons, and phenols. Amines were found only in EF plants at 15% and 30% soil moisture content.

### 3.2. Carbon Chain Length of Wax Components

**Figure 2:**
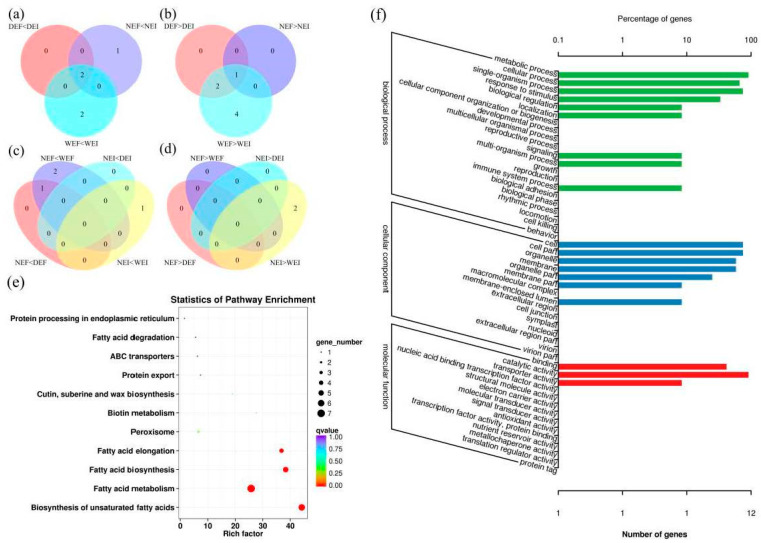
Venn diagrams comparing differentially expressed genes (DEGs) in *Achnatherum inebrians* under the status of *Epichloë gansuensis* endophyte (**a**,**b**) and different moisture content (**c**,**d**). KEGG pathway enrichment (**e**) of DEGs associated with *A. inebrians* under different moisture content and the status of *Epichloë* endophyte. The q-value ranges from 0 to 1, and a q-value closer to 0 indicates greater enrichment. Go class (**f**) of DEGs associated with *A. inebrians* under different moisture content and the status of *Epichloë* endophyte (D: drought, N: normal, W: well-watered, EI: endophyte-infected, and EF: endophyte-free).

The chain length distributions revealed a shift in chain length for several substance classes under the six treatments, including alcohols (C_11_–C_31_), aldehydes (C_11_), amines (C_8_), esters (C_19_–C_27_), ethers (C_11_ and C_21_), fatty acids (C_19_ and C_21_), hydrocarbons (C_8_–C_54_), and phenols (C_42_; Figure 2). C_31_ was the dominant alcohol. For esters, C_25_ and C_27_ were most abundant. C20 and C54 accounted for the most prominent hydrocarbon components. Furthermore, C_11_, C_8_, and C_42_ were the only aldehyde, amine, and phenol detected, respectively. C_11_ and C_21_ were the only two ethers detected in the six treatments, whereas C_19_ and C_21_ were the only two fatty acids (Supplementary Figure S1).

### 3.3. Differentially Expressed Gene (DEG) Analysis

Upon comparison with the 30% soil moisture content treatment and the EF treatment at each soil moisture content, the unigenes with gene expression fold changes greater than or equal to 2 and with an FDR value below 0.05 were defined as DEGs. Based on these strict criteria, there were five (two upregulated and three downregulated) DEGs between DEF versus DEI, four (three upregulated and one downregulated) DEGs between NEF versus NEI, 11 (four upregulated and seven downregulated) DEGs between WEF versus WEI, one (one upregulated and zero downregulated) DEG between NEF versus DEF, three (three upregulated and zero downregulated) DEGs between NEF versus WEF, zero DEGs between NEI versus DEI, and three (one upregulated and two downregulated) DEGs between NEI versus WEI. At each treatment, we detected both unique and overlapping sets of DEGs (panels a–d of Figure 2).

### 3.4. KEGG Pathway Enrichment Analysis of the DEGs

To characterize the complex biological behavior of the transcriptome, 13 DEGs were subjected to a KEGG pathway enrichment analysis (Figure 2e). The “Biosynthesis of unsaturated fatty acids (ko01040)”, “Fatty acid metabolism (ko01212)”, “Fatty acid biosynthesis (ko00061)”, and “Fatty acid elongation (ko00062)” categories were significantly enriched.

### 3.5. GO Functional-Enrichment Analysis of the DEGs

To investigate the functions of the detected 13 DEGs, GO annotation was performed. A total of 19 GO categories were assigned to the 13 DEGs (Figure 2f). GO term enrichment analysis categorized the annotated sequences into three main categories: biological process, cellular component, and molecular function. In the biological process category, “metabolic process” was the most dominant group, followed by “single-organism process” and “cellular process”. Regarding the cellular component category, “cell” and “cell part” were the dominant categories, followed by “organelle” and “membrane”. In the molecular function category, “catalytic activity” was the most dominant group, followed by “binding” and “transporter activity”.

### 3.6. Biosynthesis Pathway of Cuticular Wax

**Figure 3:**
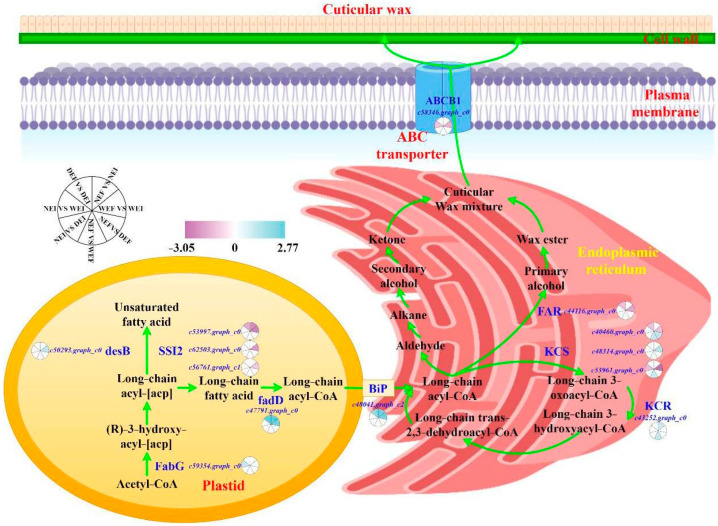
Proposed model for cuticular wax biosynthesis. Color bars ranging from purple to blue represent downregulation and upregulation in transcript expression. Gene ID is shown. Sectors of an equally divided circle represent comparisons between DEF and DEI, NEF and NEI, WEF and WEI, NEF and DEF, NEF and WEF, NEI and DEI, and NEI and WEI, respectively (D: drought, N: normal, W: well-watered, EI: endophyte-infected, and EF: endophyte-free).

A model was proposed that consists of four parts: fatty acid biosynthesis, fatty acid elongation, wax biosynthesis, and transporters (Figure 3). *FAR* (c44116.graph_c0), *KCS* (c40460.graph_c0), and *SSI2* (c53997.graph_c0) expression levels were significantly (*p* < 0.05) downregulated, whereas *BiP* (c48041.graph_c2) and *fadD* (c47791.graph_c0) expression levels were significantly (*p* < 0.05) upregulated at 15% soil moisture content in EI *A. inebrians* compared with those in EF *A. inebrians*. *SSI2* (c53997.graph_c0) expression level was significantly (*p* < 0.05) downregulated, whereas *KCR* (c43252.graph_c0), *BiP* (c48041.graph_c2), and *fadD* (c47791.graph_c0) expression levels were significantly (*p* < 0.05) upregulated at 30% soil moisture content in EI *A. inebrians* compared with those in EF *A. inebrians*. *FAR* (c44116.graph_c0), *KCS* (c40460.graph_c0, c53961.graph_c0), *SSI2* (c56761.graph_c1, c62503.graph_c0, c53997.graph_c0), and *ABCB1* (c58346.graph_c0) were significantly (*p* < 0.05) downregulated at 60% soil moisture content in EI *A. inebrians* compared with those in EF *A. inebrians*. *KCS* (c48314.graph_c0), *desB* (c50293.graph_c0), *BiP* (c48041.graph_c2), and *fadD* (c47791.graph_c0) were significantly (*p* < 0.05) upregulated at 60% soil moisture content in EI *A. inebrians* compared with those in EF *A. inebrians*. *KCS* (c40460.graph_c0) expression level was significantly (*p* < 0.05) upregulated in EF *A. inebrians* at 15% soil moisture content compared with those at 30% soil moisture content. *KCS* (c40460.graph_c0, c53961.graph_c0) and *KCR* (c43252.graph_c0) expression levels were significantly (*p* < 0.05) upregulated in EF *A. inebrians* at 60% soil moisture content compared with those at 30% soil moisture content. *KCS* (c53961.graph_c0) and *ABCB1* (c58346.graph_c0) expression levels were significantly (*p* < 0.05) downregulated, whereas *FabG* (c59354.graph_c0) expression level was significantly (*p* < 0.05) upregulated in EI *A. inebrians* at 60% soil moisture content compared with those at 30% soil moisture content.

## 4. Discussion

In the present study, we predicted that in response to different soil moisture availability, the composition of leaf cuticular wax and the expression of genes related with the cuticular wax biosynthesis pathway would change in *A. inebrians* plants host to *E. gansuensis*. Our results showed that the composition of leaf cuticular wax, especially alcohols, aldehydes, amines, and ethers, did change with different soil moisture availability, and the way these changes occurred differed with the presence or absence of *E. gansuensis.* The proportion of alcohols decreased with the increase of soil moisture in EI plants, but increased with the increase of soil moisture in EF plants. The aldehydes proportion of *A. inebrians* wax at 60% soil moisture content was significantly (*p* < 0.05) higher than that at 15% and 30% soil moisture content. Amines and ethers were only detected in the leaf cuticular wax of EF *A. inebrians* plants and EI *A. inebrians* plants, respectively. In agreement with our hypothesis, the *Epichloë* endophyte and soil moisture content changed the expression levels of 13 genes connected to cuticular wax biosynthesis and transport, such as *FabG*, *desB*, *SSI2*, *fadD*, *BiP*, *KCS*, *KAR*, *FAR*, and *ABCB1*.

Cuticular wax consistently serves a critical role in restricting nonstomatal water loss to protect the plant against environmental stresses such as drought [38]. In the present study, hydrocarbons were the major components of the cuticular wax in *A. inebrians* plants under six treatments. This was not the case in most studies. In leaves of barley and maize, the major cuticular wax components were shown to be primary alcohols, whereas alkanes comprise less than 15% of the total wax amount [39]. However, some studies also support this result. Water deficiency resulted in a four-fold rise in total wax on *Ar. thaliana* leaves, which was attributed to an overall increase of alkanes [40]. Our previous study showed that the leaf epidermal wax of EI and EF *A. inebrians* plants under different developmental stages is composed of hydrocarbons, esters, alcohols, fatty acids, phenols, and ketones, and the main group is hydrocarbon [35]. Our present study followed on from that previous study by including the interaction between drought and the *Epichloë* endophyte. Unlike the findings from *Ar. thaliana* leaves exposed to water deficiency [41,42], we did not find any DEGs associated to the hydrocarbon biosynthesis pathway. *Epichloë* endophyte greatly increased the proportion of alcohols under drought conditions, which may be closely connected to the improvement in drought resistance of EI *A. inebrians* plants.

A large number of genes encoding enzymes involved in cuticular wax biosynthesis and transporters have been identified in *Ar. thaliana* [38]. In grasses, especially maize and rice, significant progress has also been made in this area in recent years [26,31]. As nearly all *A. inebrians* plants growing in the arid, semi-arid grasslands in northwest China are host to an *Epichloë* endophyte, it is obvious that the presence of the endophyte does provide a competitive advantage under drought stress. In the present study, we used transcriptome analysis to explore the cuticular wax biosynthesis mechanism of the association of *A. inebrians* plants with *E. gansuensis* in order to clarify a potential biosynthesis model. In plastids, free fatty acids can be esterified to Coenzyme A (CoA) by long-chain-acyl-CoA synthetases (LACS), before translocation to the ER [38]. In our study, the expression level of the *fadD* gene encoding LACS was upregulated, which likely indicates that the *Epichloë* endophyte promotes fatty acid metabolism. As the first step of the acyl chain extension, the condensation of malonyl-CoA with an acyl-CoA primer is carried out by a condensing enzyme (β-ketoacyl-CoA synthase, KCS), which defines the substrate specificity of each elongation cycle [19]. Three *KCS* genes were differentially expressed in the present study. A previous study showed that OS*WSL1*, a rice homolog of Arabidopsis *KCS*, enhanced sensitivity to drought and wax-deficiency [33]. The proteins of fatty acyl-CoA reductase (FAR) that generate primary alcohols are ubiquitous in plants [18]. Our results showed that *Epichloë* endophyte downregulated the *FAR* gene expression. Once wax components reach the PM, transport across the PM into the extracellular matrix is facilitated by ABC transporters [19]. In *Ar. thaliana*, the proteins of WBC/ABCG subfamily are required for wax export to the cuticle [43]. However, we found the *ABCB1* gene of ABCB subfamily was downregulated under the well-watered condition in this study. The ABCB subfamily of genes may have a role in the drought stress response of *A. inebrians* plants with *E. gansuensis*, and be responsible for wax transport, similar to WBC/ABCG, but this remains to be further verified. Moreover, as one of the major ER chaperone proteins, binding protein (BiP) overexpression may lead to the increased tolerance to various environmental stresses of the plants by causing an ER stress response [44,45]. In the present study, *Epichloë* endophyte promoted the expression of the *BiP* gene, which may lead to a greater tolerance to stress of EI *A. inebrians* plants compared with EF *A. inebrians* plants.

In conclusion, the present study highlights that the presence of *Epichloë* endophytes can contribute significantly to the composition of cuticular wax of endophyte-symbiotic plants under different soil moisture availability. The enhanced resistance to drought of *A. inebrians* plants host to an *Epichloë* endophyte may, in part, be explained by changes in the expression levels of genes associated with the cuticular wax biosynthesis pathway. Therefore, further studies are required to verify the gene function of genes related to cuticular wax of endophyte-symbiotic plants.

## Figures and Tables

**Table 1:** Two-way ANOVA analysis showing the effects of the status of *Epichloë gansuensis* endophyte (E) and different soil moisture content (W) on the wax compositions of the leaves of *Achnatherum inebrians* (df: degree of freedom, F: F-value, and P: *p*-value).

		Alcohols		Aldehydes		Amines		Esters		Ethers		Fatty Acids		Hydrocarbons		Phenols	
Treatment	df	F	P	F	P	F	P	F	P	F	P	F	P	F	P	F	P
E	1	6.52	0.025	2.104	0.173	2.691	0.127	0.291	0.599	17.171	0.001	0.272	0.612	0.154	0.701	0.809	0.386
W	2	8.442	0.005	9.309	0.004	0.674	0.528	3.025	0.086	4.381	0.037	0.01	0.99	3.776	0.053	1.893	0.193
E × W	2	29.873	0	2.961	0.09	0.674	0.528	2.185	0.155	4.381	0.037	2.31	0.142	1.379	0.289	2.998	0.088

## Data Availability

All data supporting the findings of this study are available within the paper and within its supplementary materials published online. The RNA-seq used in this study have been deposited in the Sequence Read Achieve (SRA) of the NCBI database under the accession number PRJNA748183.

## Supplementary Materials

The following supporting information can be downloaded at: https://www.mdpi.com/article/10.3390/jof8070725/s1, Figure S1: The carbon chain length distribution detected in the cuticular wax of *Achnatherum inebrians* under the status of *Epichloë gansuensis* endophyte and different soil moisture content: alcohols (a), aldehydes (b), amines (c), esters (d), ethers (e), fatty acids (f), hydrocarbons (g) and phenols (h). Values are mean, with standard error bars (*n* = 3, D: drought, N: normal, W: well-watered, EI: endophyte-infected and EF: endophyte-free); Table S1: Qualitative GC-MS analysis of the leaf extract of endophyte-infected *Achnatherum inebrians* under 15% soil moisture content, elucidation of empirical formulas and putative identification (where possible) of the compounds; Table S2: Qualitative GC-MS analysis of the leaf extract of endophyte-free *Achnatherum inebrians* under 15% soil moisture content, elucidation of empirical formulas and putative identification (where possible) of the compounds; Table S3: Qualitative GC-MS analysis of the leaf extract of endophyte-infected *Achnatherum inebrians* under 30% soil moisture content, elucidation of empirical formulas and putative identification (where possible) of the compounds; Table S4: Qualitative GC-MS analysis of the leaf extract of endophyte-free *Achnatherum inebrians* under 30% soil moisture content, elucidation of empirical formulas and putative identification (where possible) of the compounds; Table S5: Qualitative GC-MS analysis of the leaf extract of endophyte-infected *Achnatherum inebrians* under 60% soil moisture content, elucidation of empirical formulas and putative identification (where possible) of the compounds; Table S6: Qualitative GC-MS analysis of the leaf extract of endophyte-free *Achnatherum inebrians* under 60% soil moisture content, elucidation of empirical formulas and putative identification (where possible) of the compounds; Table S7: Selected genes associated with cuticular wax biosynthesis in *Achnatherum inebrians* identified in the RNA-seq analysis.
