# Supplementary material for: Cuticular Wax Modification by Epichloë Endophyte in Achnatherum inebrians under Different Soil Moisture Availability
Source: J Fungi (Basel). 2022 Jul 12;8(7):725. doi: 10.3390/jof8070725 (PMC9325231; doi:10.3390/jof8070725)
Supplement: Supplementary file 1 [file jof-08-00725-s001.zip › Supplementary Figure.pdf]

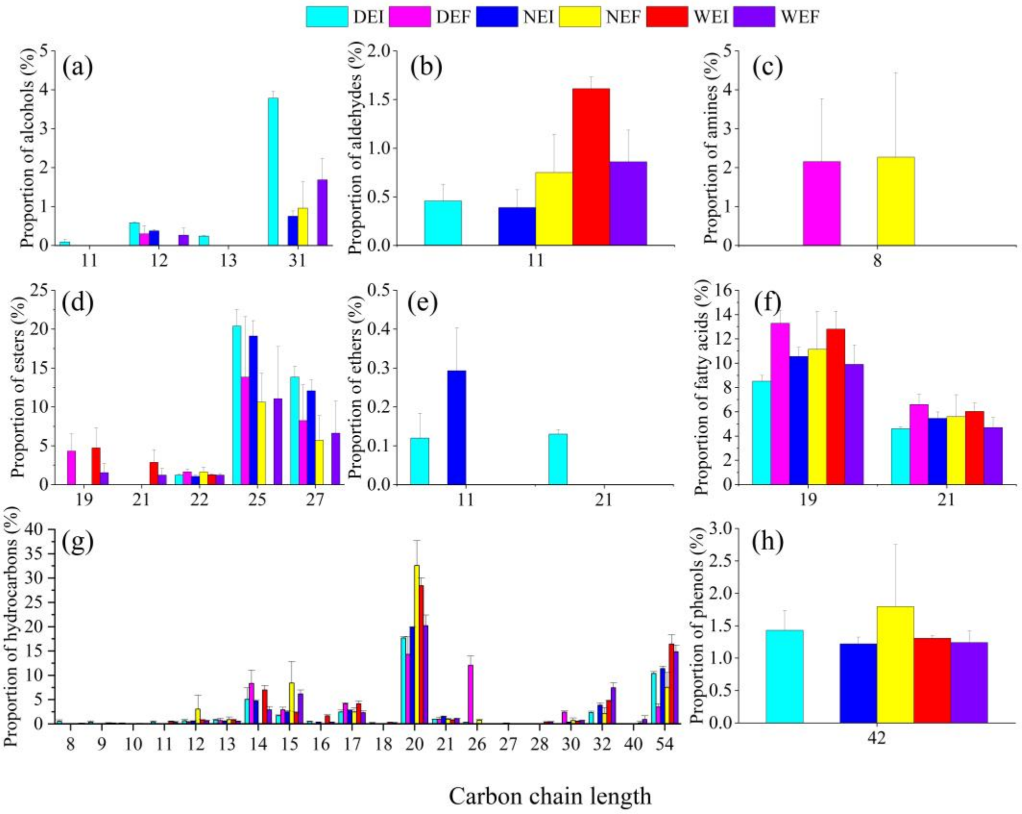

Figure S1. The carbon chain length distribution detected in the cuticular wax of *Achnatherum inebrians* under the status of *Epichloë gansuensis* endophyte and different soil moisture content: alcohols (a), aldehydes (b), amines (c), esters (d), ethers (e), fatty acids (f), hydrocarbons (g) and phenols (h). Values are mean, with standard error bars (n=3, D: drought, N: normal, W: well-watered, EI: endophyte-infected and EF: endophyte-free).
